# Surface-engineered polyethyleneimine-modified liposomes as novel carrier of siRNA and chemotherapeutics for combination treatment of drug-resistant cancers

**DOI:** 10.1080/10717544.2019.1574935

**Published:** 2019-03-31

**Authors:** Livia P. Mendes, Can Sarisozen, Ed Luther, Jiayi Pan, Vladimir P. Torchilin

**Affiliations:** a Center for Pharmaceutical Biotechnology and Nanomedicine, Northeastern University, Boston, MA, USA;; b CAPES Foundation, Ministry of Education of Brazil, Brasília, DF, Brazil;; c Department of Pharmaceutical Sciences, Northeastern University, Boston, MA, USA

**Keywords:** Drug delivery, siRNA delivery, liposomes, surface-modification, polyethyleneimine, combination cancer therapy, multidrug resistance

## Abstract

Modification of nanoparticle surfaces with PEG has been widely considered the gold standard for many years. However, PEGylation presents controversial and serious challenges including lack of functionality, hindered cellular interaction, allergic reactions, and stimulation of IgM production after repetitive dosing that accelerates blood clearance of the nanoparticles. We report the development of novel liposomal formulations surface-modified with a low molecular weight, branched polyethyleneimine (bPEI)–lipid conjugate for use as an alternative to PEG. The formulations had very good stability characteristics in ion- and protein-rich mediums. Protein adsorption onto the liposomal surface did not interfere with the cellular interaction. bPEI-modified liposomes (PEIPOS) showed enhanced association with three different cell lines by up to 75 times compared to plain or PEGylated liposomes and were without carrier toxicity. They also penetrated the deeper layers of 3D spheroids. Encapsulating paclitaxel (PTX) into PEIPOS did not change its main mechanism of action. PEIPOS complexed and intracellularly delivered siRNAs and downregulated resistance-associated proteins. Finally, tumor growth inhibition was observed in a mouse ovarian xenograft tumor model, without signs of toxicity, in animals treated with the siRNA/PTX co-loaded formulation. These complex-in-nature but simple-in-design novel liposomal formulations constitute viable and promising alternatives with added functionality to their PEGylated counterparts.

## Introduction

Over the last four decades (DeVita, 1975; DeVita et al., 1975), it has been established that when cancer treatment strategies are pursued using a single drug, the chemotherapy will eventually fail due to either severe side effects caused by the high doses of the anticancer drug, or more likely, to resistance development (Diaz et al., 2012; Bozic et al., 2013). More recently, combination treatments, especially combining chemotherapeutics with siRNA have come to represent a promising approach. However, siRNA delivery into cancer cells either alone or in combination with other drugs is a challenging task which requires overcoming unique barriers including plasma instability, very low cellular internalization and immediate degradation within the endosomal compartments of the cancer cells. In addition, various physicochemical properties of small molecule anticancer drugs and siRNAs make it very difficult to combine them and requires a versatile nanocarrier for successful applications (Krishna & Mayer, 1997, 2000; Pritchard et al., 2013; Abouzeid et al., 2014; Zuckerman & Davis 2015; Sarisozen et al., 2016).

Increasing the blood circulation time is one of the most important characteristics of a successful nanomedicine, for example, liposomes carrying any type of cargo. Polyethylene glycol (PEG), the gold standard for surface modification of nanoparticles, increases their surface hydrophilicity and creates a shield that repels the opsonizing blood proteins and other components. These ‘stealth’ characteristics ultimately increase the residence time of liposomes in the blood circulation (Salmaso & Caliceti 2013). PEG has been added to the Food and Drug Administration’s (FDA) approved nanomedicines, including DOXIL^®^ and Genexol-PM^®^ and is now being used in other nanomedicine formulations in clinical trials (Shi et al., 2017). However, PEG-coating also hinders nanoparticle interaction with cells, diminishes cellular internalization, and jeopardizes the success of transfection with oligonucleotides (Verhoef & Anchordoquy, 2013). Furthermore, careful studies have shown that repeated administration of PEG can generate IgM antibodies against it (Ishida et al., 2007) and may lead to rapid elimination of subsequent PEGylated formulations from the circulation, a phenomenon termed ‘accelerated blood clearance (ABC)’ (Abu Lila et al., 2013). PEG can also activate the complement system and provoke serious side effects such as anaphylaxis (Mima et al., 2015; Yang & Lai, 2015). Thus, it is important to find and utilize alternatives to PEG while keeping the desirable properties of PEGylation that include increased steric stability, longer blood-residence time, and effective hydrophilic surface engineering of nanoparticles.

The high positive charge density of polyethyleneimine (PEI) enables efficient condensation of negatively charged siRNA by electrostatic interactions (Godbey et al., 1999 Sonawane et al., 2003). The relatively high efficacy of PEI is, in part, due to its inherent capacity to buffer the endosomal acidic pH and disrupt the endosomal vesicles via a ‘proton-sponge effect’ (Höbel & Aigner 2013), thus providing an effective endosomal escape mechanism. However, the major drawback and limiting factor for clinical applications of PEI, especially of the high molecular weight PEIs, is its high nonspecific toxicity caused by destabilization of cellular and mitochondrial membranes and activation of intracellular apoptotic pathways (Nimesh, 2012). Low-molecular-weight PEIs have manageable cytotoxicity profiles but are far less efficient as transfection agents. Branched PEI (bPEI) of low molecular weight, on the other hand, seems to provide a favorable balance and presents lower toxicity while maintaining its moderate efficacy as a transfection agent (Kunath et al., 2003). Lipidation of bPEI through covalent attachment with dioleoyl phosphatidylethanolamine (DOPE) has been shown to further improve its gene silencing ability as an oligonucleotide carrier (Navarro et al., 2012, 2014). Equally important, bPEI has the desirable characteristics of hydrophilic polymers that are used to stabilize formulations, including flexible chains and a hydrophilic nature, which are known to provide reduced opsonization.

Here, we used a low-molecular-weight bPEI–lipid conjugate to engineer the liposome surface in attempts to develop novel nanocarrier formulations with improved stability and added functionality over non-PEGylated and PEGylated liposomes for a versatile nanomedicine platform. The possibility of co-loading siRNA along with an anticancer drug and the transfection ability of these new formulations was also investigated. We checked the efficacy on multidrug-resistant (MDR) cancer cell lines *in vitro* and *in vivo*. The simultaneous delivery of small molecule chemotherapeutics along with siRNAs that downregulate MDR-associated proteins should be especially advantageous for overcoming drug resistance and enhancing the treatment efficacy, which represents an extremely challenging task.

## Materials and methods

### Materials

Egg phosphatidylcholine (ePC), cholesterol (Chol), 1,2-dioleoyl-*sn*-glycero-3-phosphoethanolamine-*N*-(glutaryl) (NGPE) and 1,2-dipalmitoyl-*sn*-glycero-3-phosphoethanolamine-*N*-(lissamine rhodamine B sulfonyl) (Rhod-PE) were purchased from Avanti Polar Lipids (Alabaster, AL, USA). 1,2-Distearoyl-*sn*-glycero-3-phosphoethanolamine-*N*-[methoxy (polyethylene glycol)-2000] (PEG_2000_-PE) was acquired from Corden Pharma International (Plankstadt, Germany). Branched polyethyleneimine (bPEI) with a molecular weight of 600 Da (confirmed with MALDI-TOF) was purchased from Polysciences, Inc. (PA, USA) and paclitaxel (PTX) was from LC Laboratories (Woburn, MA). Human cancer cell lines of HeLa (cervical) were purchased from ATCC (Manassas, VA) and A2780-ADR (adriamycin-resistant ovarian carcinoma) from Sigma (ECACC, UK). The paclitaxel-resistant human ovarian cancer cell line, SKOV-3TR, was a kind gift from Dr. Duan Zhenfeng (MGH, Boston, MA). CellTiter-Blue^®^ cell viability assay was obtained from Promega Corp. (Madison, WI). Hochest33342, Yo-Pro-1-iodide (YoPro), and propidium iodide (PI) were purchased from Life Technologies (Carlsbad, CA). siRNA targeting MDR1 (siMDR1): 5′- GGAAAAGAAACCAACUGUCdTdT-3′ (sense) and siRNA nontargeting (siSCR) duplex: 5′-AGUACUGCUUACGAUACGGdTdT-3′ (sense) were purchased from Dharmacon (CO, USA). Phycoerythrin-conjugated anti-P-glycoprotein antibody [UIC2] was purchased from Abcam (Cambridge, MA). All other reagents were of analytical grade.

### Synthesis of the polyethyleneimine–phospholipid conjugate (bPEI-PE)

The bPEI-PE conjugate used to coat the liposomes was synthesized following the method in Sawant et al. (2012) with slight modifications. Briefly, *N*-Hydroxysuccinimide (NHS) (88 µmol) and ethyl(dimethyl aminopropyl) carbodiimide (EDC) (86 µmol) were solubilized in methanol and added to a chloroform solution containing NGPE (8 µmol) under magnetic stirring, for 1 hour at room temperature. BPEI (27 µmol) was solubilized in chloroform, added dropwise to the activated NGPE under magnetic stirring, and agitation was maintained overnight at room temperature. The organic solvent was removed by rotary evaporation; conjugate was resuspended in deionized water and purified from free bPEI by dialysis (MWCO 3.5 kDa) against excess deionized water. The purified solution was lyophilized, resuspended in chloroform and stored at −80 °C. The synthesized conjugate was characterized by thin-layer chromatography (TLC).

### Preparation and characterization of liposomes

PTX-loaded liposomes (1.5% to 4% *w*/*w*) were prepared using ePC and Chol (90:10 mol%) by the thin-film formation method followed by extrusion. Briefly, ePC, Chol and PTX were solubilized in a chloroform/methanol mixture, and the organic solvents were removed to form the thin lipid film. The film was rehydrated with phosphate buffer saline (PBS) pH 7.4 at a 10 mg/mL concentration of lipids, and extruded 15 times through 100 nm polycarbonate membranes. bPEI-PE films were also prepared by solubilizing the conjugate in chloroform, which was evaporated to form a thin film. After extrusion, liposomes were added to bPEI-PE films at the mol ratios of 0.1% and 0.5% (mol% of bPEI-PE:liposomal lipids). Liposome formulations were incubated at 37 °C under mild agitation for 4 hours for stable postinsertion of bPEI-PE into the lipid bilayer. Noncoated (plain) and coated liposomes were filtered through 0.22 µm PES membranes for sterilization purposes as well as separation of the nonencapsulated drug. To prepare fluorescently labeled liposomes, Rhod-PE was added in the preparation of the lipid film at a final concentration of 0.1% (%mol of total lipids). Plain liposomes were named 0% PEIPOS and coated ones were named as 0.1% PEIPOS or 0.5% PEIPOS depending on the mol% of bPEI-PE used in the coating step. After filtration, PEIPOS were characterized by size, polydispersity index (PdI) and zeta potential (ZP) by dynamic light scattering (DLS) in a Zetasizer Nano ZS 90 (Malvern Instruments, UK), with the samples previously diluted with ultrapure water. Encapsulation efficiency was determined by a validated reversed-phase HPLC method using a Hitachi Elite LaChrome HPLC system equipped with an auto-sampler (Pleasanton, CA) and diode array detector (Sarisozen et al., 2014).

### *In vitro* drug release

For the *in vitro* release studies, the acceptor media were prepared with PBS containing 1% (*w*/*v*) sodium dodecyl sulfate (SDS) at pH 5.0 and 7.4. A volume of 500 μl of either PEIPOS containing PTX or free PTX solubilized in the release media was added to a Float-A-Lyzer^®^ G2 ready-to-use dialysis device (MWCO 50 kD). Since PTX is not soluble in PBS alone (formulation buffer), the drug was solubilized in the release media (1% SDS *w*/*v* in PBS), which allowed using an equal initial concentration of PTX for both the liposomes and the free form. The devices were immersed in 13 mL of each acceptor media and maintained in an orbital shaker at 37 °C at 125 rpm for 48 h. Samples were withdrawn from the release media and replaced with fresh media at predetermined time points over 48 h. The amount of drug released in the acceptor compartment was analyzed by HPLC after dilution with the mobile phase. The assay was performed in triplicate, and sink conditions were maintained during the experiment. Samples were then filtered through a 0.45-μm membrane, diluted in the mobile phase and analyzed by HPLC. To ensure sink conditions, the solubility of the drug was assessed in PBS containing 1% SDS for 24 h at 37 °C under agitation. The solubility of PTX in these media was determined as 200 μg/mL.

### Stability of PEIPOS in fetal bovine serum

Noncoated, 0.1% and 0.5% coated PEIPOS were incubated in 10% (*v*/*v*) fetal bovine serum (FBS) containing PBS at 37 °C under mild agitation (150 rpm on a horizontal shaker). At predetermined time points of 1, 4 and 24 h, the changes in size and ZP were measured as described earlier. All samples were incubated at a concentration of 500 µg/mL of lipids and diluted with ultrapure water for the measurements.

### Analysis of PEIPOS-associated proteins

To evaluate protein adsorption onto the surface of the liposomes, noncoated (0%) and 0.5% PEIPOS at 1 mg/mL of lipid concentration were incubated in 10% (*v*/*v*) FBS containing PBS pH 7.4 overnight at 37 °C under mild agitation. Liposomes of ePC and Chol (90:10 mol%) coated with PEG_2000_-PE (3 mol%) were also prepared in the same way as PEIPOS for this assay. After incubation, samples were submitted to ultrafiltration in 300 kDa MWCO filters and centrifuged at 4000 *g* for 10 minutes to separate free and PEIPOS-associated proteins. The proteins were analyzed by SDS-PAGE under nonreducing conditions after lysing the liposomes with RIPA^®^ buffer (Thermo Scientific, Waltham, MA). The run was performed at 225 V for 40 minutes in polyacrylamide/bis-acrylamide gradient gel (4–20%) using prestained SDS-PAGE molecular weight standards (Thermo Scientific Spectra Multicolor Broad Range Protein Ladder) to estimate the molecular weight of the proteins adsorbed on to the PEIPOS. After the run, the gel was stained with GelCode^®^ Blue Stain Reagent (Thermo Scientific, Waltham, MA) to allow visualization of the protein bands.

### Cell culture and spheroid preparation

The human cervical cancer cell line HeLa, the PTX-resistant human ovarian adenocarcinoma cell line SKOV3-TR, the adriamycin-resistant human ovarian carcinoma cell line A2780-ADR and its sensitive counterpart, A2780, were allowed to grow in complete RPMI cell culture medium supplemented with 10% FBS (*v*/*v*), 50 U/mL penicillin, 50 µg/mL streptomycin and 2 g/L glucose. The A2780-ADR cell line was exposed to 100 nM doxorubicin twice a week for 48 h, as recommended by the supplier. All cell lines were incubated at 37 °C, in a 5% CO_2_ atmosphere and high humidity.

Multicellular tumor cell spheroids of HeLa cells were prepared by the liquid overlay method as described previously (Sriraman et al., 2016). The cells were seeded on agar-coated 96-well plates at 10,000 cells/well in 100 µl complete medium. The plates were then centrifuged for 15 min at 1500 rcf at 20 °C. Spheroids formed 3–5 days after seeding were used under the same conditions as monolayers.

### Cell association and internalization with monolayers

HeLa, SKOV3-TR and A2780-ADR cells were used to evaluate the association of the formulations with these cell lines in monolayers. Briefly, the cells were seeded at a density of 100,000 cells/well in 12-well plates 24 h prior to the experiment and then treated with Rhod-PE labeled 0%, 0.1%, and 0.5% PEIPOS in serum-complete medium. The final lipid concentration for the treatments was 100 µg/mL for all cell lines. HeLa cells were also treated with a low lipid concentration of 10 µg/mL to investigate the liposome count:cell number effect. After 4 h of incubation at 37 °C, the cells were harvested, washed and immediately analyzed by a FACSCalibur flow cytometer (Beckton Dickinson, Franklin Lakes, NJ) using a 488 nm blue laser for excitation and FL2 channel (585/42 nm wavelength filter) for recording the rhodamine fluorescence intensity. Cells were gated to exclude debris and dead cells, and 10,000 events were collected for each sample (*n* = 3). Mean fluorescence intensity (MFI) of the cells was compared to a control group treated with non-labeled PEIPOS.

To investigate whether the cellular entry was driven by surface charge or energy-dependent mechanisms, A2780-ADR cells were seeded in 12-well plates at 80,000 cells/well 24 h before the assay. The cells were treated with Rhod-PE-labeled liposomes in serum-complete medium for 90 min at either 37 °C or 4 °C. An additional pretreatment group was included where cells were pre-incubated with bPEI at 10 µg/mL for 15 min. At the end of treatment, the cells were washed with PBS, harvested by trypsinization, washed again twice and analyzed by flow cytometry. PEGylated liposomes were also used as a comparison group in this assay.

### 3 D Spheroid association and penetration

HeLa spheroids were treated for 4 h with Rhod-PE labeled 0%, 0.1%, and 0.5% PEIPOS formulations 5 days after seeding. Lipid concentration was kept at 100 µg/mL, and five spheroids were used as one replicate. Treatments were done in triplicates (total of 15 spheroids/formulation). For flow cytometry analysis, spheroids were washed twice with PBS and dispersed into single cells by 10 min incubation with AccuMax^®^ cell detachment/disassociation solution at 37 °C (Sarisozen et al., 2016). The cell detachment solution was inactivated by FBS addition and cells were centrifuged, washed and redispersed in PBS and immediately analyzed by flow cytometry using the settings described earlier.

To investigate the liposome distribution in the spheroids by confocal laser scanning microscopy (CLSM), the spheroids were treated with the formulations the same way as in flow cytometry studies. Following treatments, the spheroids were washed with PBS and fixed in 4% paraformaldehyde overnight at 4 °C. After washing with PBS, spheroids were placed in 16-well glass chamber slides (Nunc™ Lab-Tek™ Chamber Slide System, USA), and the analysis was performed with a Zeiss LSM 700 confocal microscope. The spheroids were imaged at 10 µm Z-stack intervals starting from the apex of the spheroids using a 555 nm laser and 10x objective. The images were analyzed using Image-J software 1.51n (NIH, Bethesda, MD) with Fiji package (version 1.0) (Schindelin et al., 2012) to evaluate the penetration profiles. A circular area with the same diameter and location indicating the core of the spheroids was selected from every optical section. The relative area of selection was kept constant among different spheroids. The corrected integrated pixel densities in the selection area were plotted against the section distance from the spheroid apex to create the profiles. Z-projections using maximum pixel intensity were also created to better reflect the differences among the overall penetration profiles.

### *In vitro* cytotoxicity on monolayers

To evaluate *in vitro* cytotoxicity, drug-sensitive HeLa and multidrug-resistant A2780-ADR cells were seeded in 96-well plates at a density of 3000 cells/well 24 h before treatment. Different formulations were added to the cells at various concentrations, ranging from 0.3125 nM to 10 nM for HeLa cells and 1 µM to 32 µM for A2780-ADR cells. Cells were treated either continuously for 48 h in serum-complete medium (HeLa) or treated for 4 h followed by 44-h incubation in complete fresh medium (A2780-ADR). Cell viability at the end of the treatments was assessed with CellTiter-Blue^®^ assay following the manufacturer’s protocol (Promega Corporation, Fitchburg, WI). Fluorescence intensity was recorded (excitation 560 nm, emission 590 nm) in a microplate reader (BioTek, Model EL800, Winooski, VT). The viability of the treated cells was compared to a nontreated control.

### β-tubulin immunostaining

To confirm that the main mechanism of action of PTX was a microtubule-stabilizing effect of PTX and whether it was affected by PEIPOS-mediated delivery, immunofluorescent imaging studies were conducted. Briefly, HeLa cells were seeded on glass coverslips in 12-well plates at 100,000 cells/well one day before treatment. Cells were treated with the free drug, 0% and 0.5% PEIPOS at 2.5 nM of PTX concentration and incubated for 24 h continuously at 37 °C in complete media. After that, cells were fixed with 2% paraformaldehyde at room temperature for 15 mins, permeabilized for 5 min with cold methanol and incubated with PBS containing 3% bovine serum albumin (BSA) (*w*/*v*) at room temperature for 30 mins to block nonspecific protein interaction. Next, cells were incubated overnight at 4 °C with a 1:20 dilution of primary anti-β-tubulin antibody (mouse monoclonal to β-tubulin, G-8, sc-55529, Santa Cruz Biotechnology, Dallas, TX). The primary antibody solution was removed, coverslips were washed thrice, and the FITC-labeled secondary goat anti-mouse IgG (Santa Cruz Biotechnology, Dallas, TX) was used at a 1:30 dilution to incubate the cells for 2 h at room temperature. All antibody dilutions and staining were performed in blocking buffer. Finally, cells were washed with PBS, their nuclei stained with 5 µM Hoechst 33342, and coverslips were mounted on slides for CLSM analysis.

### Laser scanning cytometry

HeLa cells were seeded at 3000 cells/well in black-walled optical-bottom 96-well plates one day before the experiment. Cells were treated with the free drug, 0%, or 0.5% PEIPOS at different concentrations for 48 h. Next, Hoechst 33342, YoPro and PI were diluted with medium and added directly to the wells at 5, 0.12, and 1 µg/mL final concentrations, respectively. Stained cells were analyzed *in situ* with an iCyte imaging cytometer (Compucyte Corp., Westwood, MA). Hoechst was excited with a 405 nm laser, and the fluorescent signal was collected through a 440/30 nm bandpass filter. YoPro and PI were excited by a 488 nm laser and fluorescent signals collected at 515/30 nm bandwidth and from a 650 nm long pass filter, respectively. The nuclei stained by Hoechst allowed the quantification of total cellular DNA content and, based on nuclear area, single live cells were gated into G1, S, and G2 phases to categorize the cell cycle distribution. Quantification of apoptotic events was done based on cell morphology and by random segmentation by gating the cells based on YoPro fluorescence, used to identify early apoptosis events, and PI fluorescence, which stains for late apoptosis/necrosis.

### Complexation of PEIPOS with siRNA, gel retardation, size and zeta potential changes

The complexation of PEIPOS with siRNA was monitored by gel retardation electrophoresis. bPEI-coated PEIPOS, 0.5% (by moles), was used to complex siSCR at different ratios of nitrogen/phosphate (N/P), present in PEI-PE and siRNA, respectively. The complexes were prepared with a fixed amount of siRNA diluted in 5% glucose (*w*/*v*) buffered HEPES pH 7.4 (BHG) and varying amounts of liposomes diluted with the same solution, mixed in equal volumes and incubated for 15 min. Nucleic acid (1 µg) was loaded onto a 0.8% agarose gel, and electrophoresis was performed using an E-Gel electrophoresis system (Life Technologies, Carlsbad, CA), followed by evaluation of the bands under UV light. Size and zeta potential of the complexes were also verified to check for changes of these parameters after complexation with siRNA.

### Characterization of P-glycoprotein overexpression of cells

A2780-ADR cells and their drug-sensitive A2780 counterparts were seeded in 6-well plates at 500,000 cells/well to characterize their P-glycoprotein (P-gp) expression patterns. The cells were detached on the next day with the aid of enzyme-free cell disassociation buffer (EMD Millipore), washed twice with 2% BSA (*w*/*v*) in PBS pH 7.4 containing 0.1% (*w*/*v*) sodium azide (staining buffer) and incubated for 45 minutes on ice with phycoerythrin-labeled anti-P-glycoprotein antibody (UIC2, Abcam). Cells were then washed twice with staining buffer and resuspended in this same solution for immediate flow cytometer analysis. An isotype matching control was used to evaluate the nonspecific antibody binding and to normalize the results.

### siRNA-mediated P-gp downregulation

Transfection efficiency of PEIPOS/siMDR1 complexes was evaluated in P-gp overexpressing A2780-ADR cells. Cells were seeded into 6-well plates at 100,000 cells/well. Complexes were prepared at an N/P of 13, and cells were treated with 100 nM of siMDR1/well for 4 h at 37 °C in serum-complete medium, one day after seeding. Cells were washed gently and incubated for an additional 44 h at the same conditions. The cells were then detached using enzyme-free cell dissociation buffer and processed using the same protocol described earlier for P-gp staining. bPEI-PE polymeric micelles previously developed and characterized in our laboratory (Navarro et al., 2012) complexed with siMDR1 at N/P of 16 were used as a positive control. Samples were analyzed by flow cytometry, and fluorescence intensity of treated cells was compared to a P-gp stained nontreated control.

### *In vivo* animal model

The experiments were performed in 5- to 6-week-old female athymic nude mice following protocol 16-1134 R approved by Northeastern University Institutional Animal Care and Use Committee. The animals were obtained from Charles River (Wilmington, MA) and weighed between 22 g and 25 g throughout the study. They were provided food and water *ad libitum*. After the acclimatization period of 7 days, the animals were inoculated subcutaneously near the right flank with two million A2780-ADR cells in 100 µL of sterile PBS pH 7.4.

### *In vivo* evaluation of the efficacy of the nanopreparations

For the tumor inhibition assay, when tumors reached approximately 150 mm^3^, animals were randomly separated into 5 groups (*n* = 4). The different treatments administered IV, via the tail vein, were: (a) PBS Ph 7.4 as control; (b) Free paclitaxel; (c) 0.5% PEIPOS/PTX; (d) 0.5% PEIPOS/PTX/siMDR1. The treatment regimen consisted of IV injections every other day containing 5.5 mg/kg of PTX, and 0.8 mg/kg of siMDR1 for up to 12 injections or until tumor reached 1500 mm^3^, when animals were then euthanized. The tumor sizes were monitored by measuring with the aid of a Vernier caliper, every other day, beginning from the first day of injection until the day the animals are euthanized. Tumor volume was calculated based on the formula: *V* = 0.5 × W × W×L, where *W* is the width (smaller dimension of the tumor), and *L* is the length (the largest dimension). At the end of the experiment, animals were euthanized, tumors were collected, weighed, and snap-frozen with liquid nitrogen for further analysis. Blood was also collected via the cardiac puncture method from all the mice and centrifuged at 2000 *g* for 30 min at 4 °C to separate the plasma, which was then stored at −80 °C for further analysis of toxicity.

### Toxicity evaluation of PEIPOS in mice

The toxicity of the formulations used was evaluated based on a change in body weight of the animals along the treatment. For that, animals were weighed every other day, beginning from the first day of injection until the day the animals were euthanized. Alanine aminotransferase (ALT) levels were measured in the plasma using a colorimetric assay kit following the manufacturer’s instructions (Abcam, Cambridge, MA).

### Statistical analysis

Experiments were performed in triplicate, and results were expressed as mean ± standard deviation. Unless otherwise indicated, analysis of variance test (ANOVA) followed by Tukey’s multiple comparisons test was used for comparison of differences between groups. Statistical difference was accepted when *p* < .05. Statistical analysis was performed using GraphPad Prism software (version 7.0a for Mac OS, GraphPad Software, La Jolla, CA).

## Results

### Surface engineering with bPEI increases the stability of liposomes

The in-house synthesized amphiphilic bPEI-PE conjugate obtained by the reaction of the NHS-activated carboxyl group in NGPE and an amino group in bPEI (Sawant et al., 2012) was used to coat plain liposomes (0%PEIPOS) at 0.1% and 0.5% by moles (0.1%PEIPOS and 0.5%PEIPOS, respectively) (Table 1).

**Table 1.** Physicochemical characteristics of noncoated and coated liposomes.

Formulation	Mean diameter (nm)	PdI	Zeta potential (mV)	Concentration of PTX (µM)	Encapsulation efficiency (%)	Retained drug (%)^a^
0% PEIPOS	155 ± 9.2	0.08 ± 0.02	−4.3 ± 1.5	132.3 ± 19.1	75.3 ± 11.2	74.7 ± 13.1
0.1% PEIPOS	163 ± 4.8	0.12 ± 0.03	+1.9 ± 0.2	151.3 ± 6.5	86.0 ± 4.0	94.0 ± 9.5
0.5% PEIPOS	161 ± 9.4	0.08 ± 0.01	+13.7 ± 2.3	147.7 ± 7.0	85.3 ± 3.2	97.7 ± 1.5

Results express the average of three independent batches prepared with 1.5% PTX (*w*/*w*)±SD.

a Drug retained encapsulated in the liposomes was evaluated after storage of liposomes for seven days at 4 °C.

**Figure 1.**
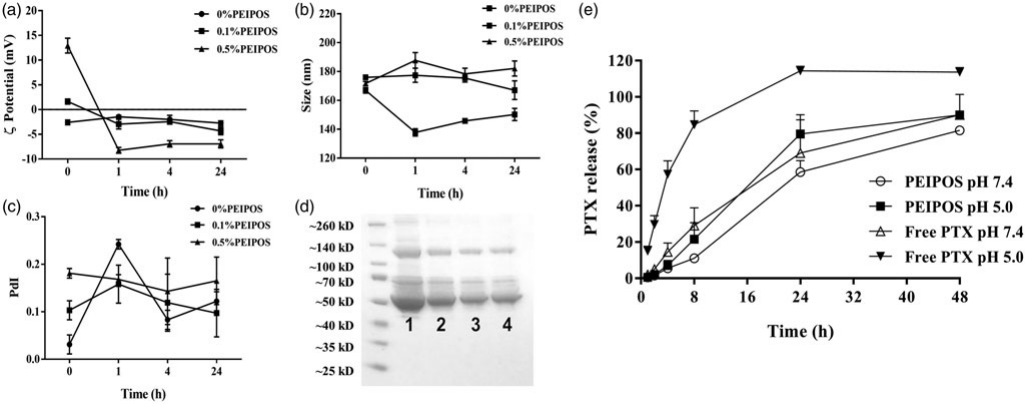
Physical–chemical characterization of PEIPOS. (a–d) Stability of PEIPOS upon incubation in 10% (*v*/*v*) FBS in PBS pH 7.4 at 37 °C for over 24h. (a) ZP (mV), (b) particle size (nm), and (c) PdI values at given time points. Error bars represent the standard deviation of three independent sample measurements. (d) Proteins adsorbed in liposomes incubated in 10% FBS at 37 °C overnight. Lane 1 shows FBS diluted to 2.5% (*v*/*v*), lanes 2 and 3 are 0% PEIPOS and 0.5% PEIPOS and lane 4 is a PEGylated liposomal formulation (3 mol% PEG2000-PE). (e) Cumulative in vitro release of PTX from bPEI-coated liposomes at different pH under sink conditions. Free PTX groups were used as controls to evaluate the diffusion rate through the release membrane.

Liposomes were incubated in PBS with 10% (*v*/*v*) of FBS to various time-points at 37 °C to mimic the *in vivo* salt and serum protein concentrations (Gaspar et al., 2014). Results show that the size of bPEI-coated liposomes did not change significantly (Figure 1(b)). The changes in coated-liposome PdI values were also moderate (Figure 1(c)). This was in stark contrast with the noncoated liposomes; where, both particle size and especially the PdI changes were significant. Regardless of the surface modification with bPEI and the initial zeta potential (ZP) values before serum incubation, the ZP of all samples decreased to a value of approximately −5 mV (Figure 1(a)). The sharp decrease in ZP of 0.5%PEIPOS is consistent with the adsorption of serum proteins, mainly albumin (66 kDa) and globulins (140 kDa), (Dos Santos et al., 2007) onto the surface of the liposomes (Figure 1(d)). The results show that the profile of proteins adsorbed onto PEGylated liposomes (Figure 1(d), lane 4) is the same as on 0% and 0.5% PEIPOS, but to a lesser extent. When the band intensities were evaluated by Fiji software, it was found that, compared to noncoated liposomes, 0.5%PEIPOS had 30% less protein adsorbed on its surface, similar to PEGylated liposomes (Supplementary Figure S1). Furthermore, almost all the hydrophobic drug remained in the bilayer of the liposomes after one week of storage (Table 1). These results indicate that the surface modification of the liposomes with bPEI imparts higher stability to the nanoformulations in serum-containing media and helps to prevent drug leakage.

### *In vitro* drug release from bPEI-coated liposomes

The dialysis method was used to assess the *in vitro* release of PTX from 0.5%PEIPOS, which was measured at different time-points and at pH 7.4 and pH 5.0 along 48 h. As can be seen from Figure 1(e), the release from 0.5%PEIPOS at physiological pH was significantly lower than any of the tested groups with only a 10% cumulative release of the PTX. More than 80% of the drug was released from the 0.5%PEIPOS at pH 7.4 after 48 h. At a pH of 5.0, released PTX amounts were 22% and 90% after 8 h and 48 h, respectively, indicating a pH-responsive release behavior.

### bPEI-coated PEIPOS improve uptake in monolayers and penetration in a 3D spheroid model

**Figure 2.**
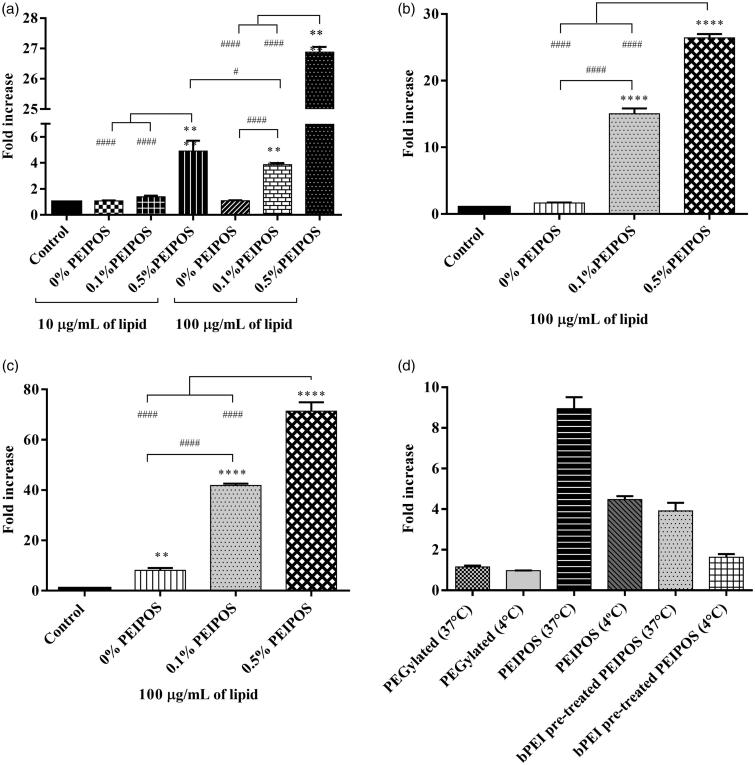
Cellular association of coated and noncoated PEIPOS with monolayers of three different cancer cell lines; (a) HeLa, (b) A2780-ADR, and (c) SKOV-3TR. Lipid concentration used for each cell line is indicated below each graph and incubation time was 4h. Control groups are nontreated cells. * indicates difference from control, whereas # indicates differences within the groups. Mean ± SD, *n* = 3, **p < .001, ****p < .0001. One-way ANOVA with Tukey’s multiple comparisons test. (d) Mode of internalization by A2780-ADR cells by flow cytometry. Lipid concentration was 100 µg/mL and incubation time was 90 min. PEGylated liposomes were modified with 3 mol% PEG2000-PE. Results are given as the fold-increase over the nonfluorescently labeled control formulations.

Cell association was assessed in HeLa, SKOV3-TR, and A2780-ADR cells. The analysis was performed using complete media, where proteins get adsorbed onto the surface of liposomes and decrease the ZP (Figure 1(a)). Even with this shift in ZP, the interaction with all cell lines was augmented after liposomes were coated with bPEI (Figure 2(a–c)). In HeLa cells, 0.5%PEIPOS showed the highest cellular association, with a 5-fold increase compared to noncoated liposomes, even when a low concentration of lipids (10 µg/mL) was used. At 100 µg/mL lipids, the cell interaction with 0.5%PEIPOS was 27-fold and 7-fold higher than control and 0.1%PEIPOS, respectively. In A2780-ADR and SKOV-3TR MDR cells, results indicate a similar trend.

To further characterize and to confirm the mode of entry of liposomes into the cancer cells, we performed a cellular association study of the PEIPOS formulations at 37 °C and 4 °C (Figure 2(d)). The results clearly laid out the challenges to PEGylation, which had the lowest cellular association at both temperature levels. When the cells were incubated with PEIPOS at 4 °C, the active transport (endocytosis) mechanisms were inhibited based on the observation of a significant decrease in association. However, compared to the control, PEIPOS still yielded a 4-fold increase in association at low temperature, indicating that some of the interaction might still be charge driven. The pretreatment of the cells with free bPEI prior to formulation decreased the overall association at both temperatures, suggesting the saturation of charge-based interactions. The same level of association was achieved when the cells were either incubated at 4 °C or pretreated with bPEI, indicating that more than 50% of the cellular internalization was driven by receptor-based interactions, not the positive-charge driven internalization.

**Figure 3.**
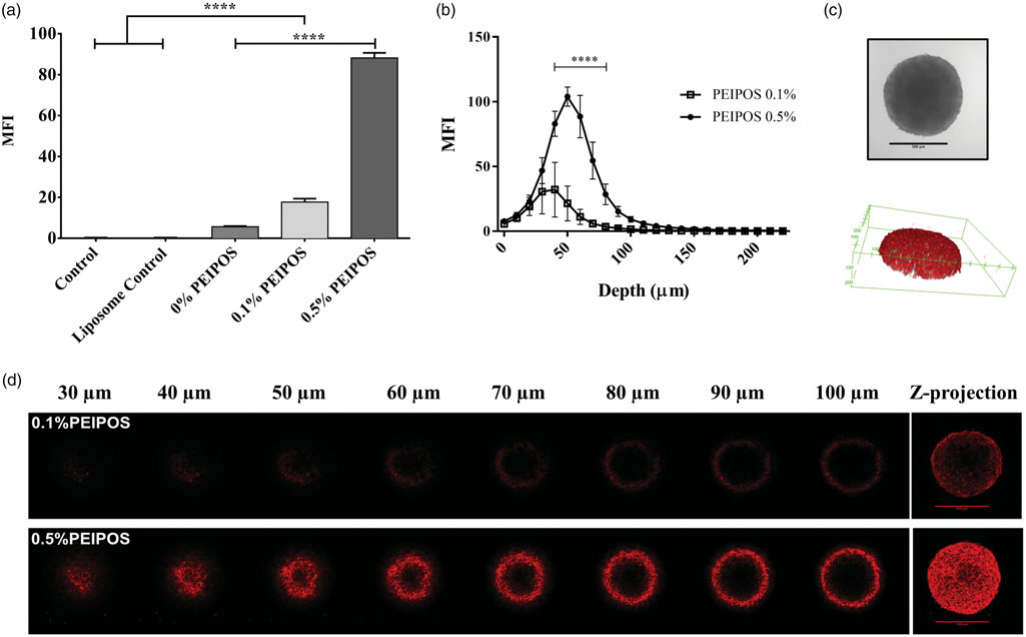
Kinetics of cellular association and penetration of formulations in 3D HeLa spheroids. (a) Rhodamine intensity increase as the indicator of cellular association of liposomes with HeLa cells after disassociation of spheroids into single cells, obtained by flow cytometry analysis. Control groups are nontreated cells, while control liposomes are cells treated with nonlabeled liposomes. *n* = 3, a total of 15 spheroids, mean ± SD, one-way ANOVA with Tukey’s multiple comparison tests, ****p < .0001. (b) Corrected integrated pixel density values of rhodamine vs. optical section depth as a representation of liposome distribution throughout the spheroids. *n* = 5, mean ± SD, two-way ANOVA with Sidak’s multiple comparisons, ****p < .0001. (c) A representative HeLa 3D spheroid incubated with 0.5% PEIPOS formulation, imaged by CLSM PMT. Scale bar indicates 500 µm. The 3D reconstruction of the same spheroid using rhodamine intensity confirmed the spheroidal shape of the 3D cell model. (d) Rhodamine-labeled liposome penetration into the spheroids at different layers of depth. The Z-projection was obtained using maximum pixel intensity collected from each layer of the spheroid. MFI, mean fluorescence intensity. Scale bars = 500 µm.

Cellular association of formulations with the cancer cells in a 3D structural model organization that closely reflect the solid tumors (Mehta et al., 2012; Zanoni et al., 2016) was investigated by analyzing individual cells forming spheroids by flow cytometry. Compared to control groups, 0.5%PEIPOS association increased 88% (Figure 3(a)). It should be noted that flow cytometry analysis involves all cells of the spheroid structure. Thus, confocal laser scanning microscopy (CLSM) studies were conducted to investigate the penetration ability of formulations into an intact 3D tumor mimicking model. PEIPOS 0.1% penetrated the spheroid structure. The maximum fluorescent signal was collected at a 40 μm depth from the apex of the spheroid (Figure 3(b,d)). However, the signal from 0.5%PEIPOS was significantly higher in layers starting from 30 μm, with the difference of approximately 75% at 50 μm. The results clearly indicate that 0.5%PEIPOS can penetrate deeper into a spheroid structure by inter- and intra-cellular routes (Figure 3(b,d)).

### bPEI-coating enhances the *in vitro* cytotoxicity of liposomes by inducing β-tubulin polymerization and subsequent apoptosis

**Figure 4.**
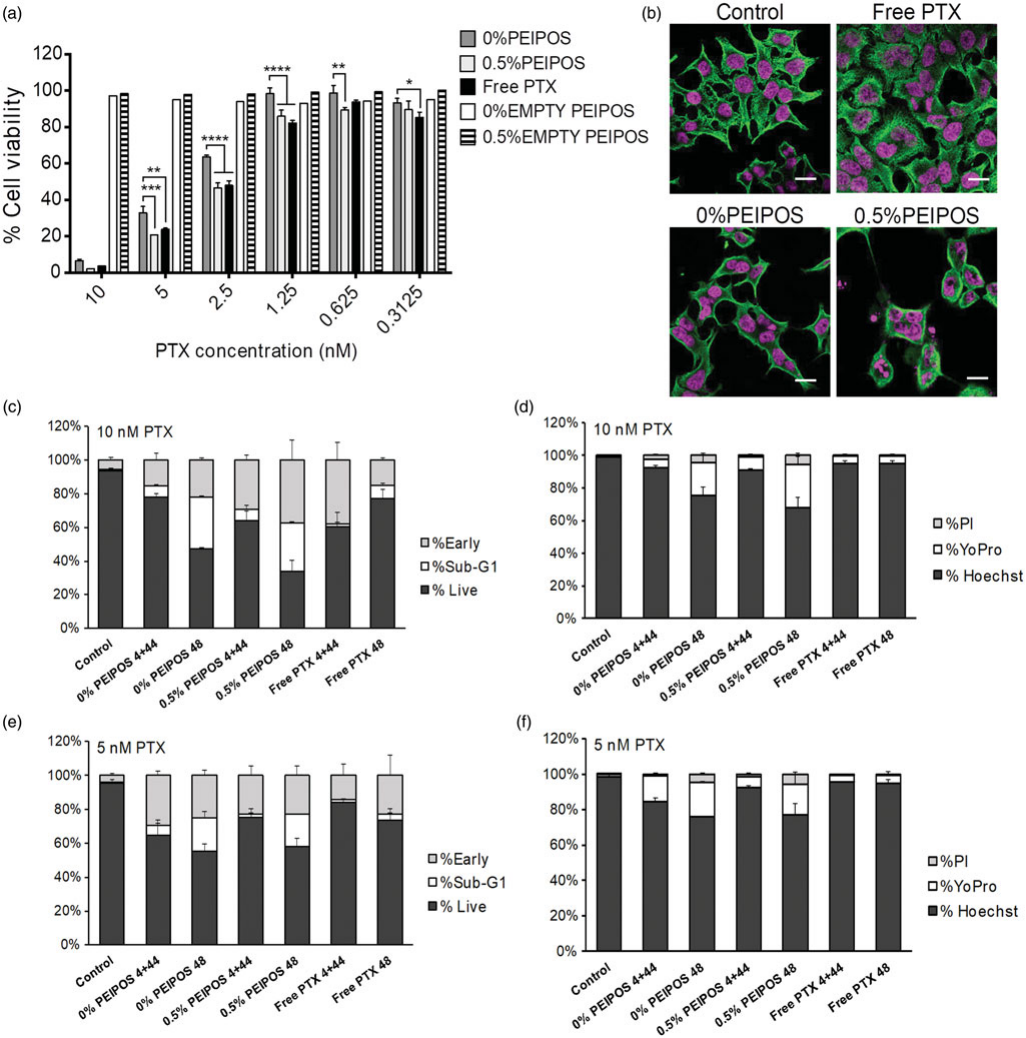
Cell viability and investigation of PTX course of action on HeLa cells. (a) Cytotoxicity profile of HeLa cells after continuous treatment with the formulations for 48h or 4 + 44h in serum-complete medium. Empty PEIPOS consists of formulations without PTX used at the same lipid concentration as those containing PTX. Data shown indicate triplicate mean ± SD from a blinded experiment. *p < .05, ***p < .0005, ****p < .0001, two-way ANOVA with Tukey’s multiple comparisons test. (b) Immunofluorescent detection of liposomal PTX-mediated? -tubulin polymerization on HeLa cells. Nuclei of the cells were stained with Hoechst (magenta), and? -tubulin structures stained green. (c-f) LSC analysis of HeLa cells treated with different formulations and for different time points. (c) and (d) Cell cycle distribution depending on the gating outlined in figure S2a for different time points, treatments, and PTX concentrations. (e) and (f) Analysis of the cells at their different stages indicated by the nuclei staining with Hoechst, early apoptotic cell staining with YoPro and necrotic cell staining with PI. Scale bars = 20 µm.

The cytotoxicity evaluation of HeLa cells with the different bPEI-coating showed enhanced liposomal PTX cytotoxicity up to the levels that matched the free drug (Figure 4(a)). The PTX IC_50_ of 0.5%PEIPOS was 2.4 nM compared to 2.5 nM for free PTX. The IC_50_ improvement by bPEI-surface modification was 50% compared to that for 0%PEIPOS of 3.8 nM, and no cytotoxicity was observed for empty PEIPOS.

Immunofluorescent analysis of β-tubulin showed that free PTX-treated cells presented microtubules with thicker structures, an indication of their stabilization, whereas untreated cells had a microtubule organization with fine and diffuse microtubule structure (Figure 4(b)). For the liposomal formulations, especially for 0.5%PEIPOS, the greater effect of this stabilization with polymerization of tubulins around the nuclei caused the cells to round up. Cells normally proceed toward mitosis, but tubulin stabilization leads to cell cycle arrest, with cells becoming multinucleated (Zhang et al., 2016).

Whether or not this microtubule polymerizing capacity of PTX caused subsequent apoptosis was also investigated using imaging laser scanning cytometry (LSC) analysis in a single end-point experiment (Figure 4(c–f)). HeLa cells were either treated 48 h continuously (indicated as 48) or treated for 4 h, followed by 44 h of incubation after drug removal (indicated as 4 + 44). By using LSC analysis based on Hoechst-stained DNA content and cell morphology, cells were gated and classified as live, sub-G1 on early apoptotic cells (Supplementary Figure S2(a)). We observed substantially more cells in sub-G1 for 0% and 0.5%PEIPOS after the 48 h continuous regimen when compared to the other treatments (Figure 4(c)). The highest number of early apoptotic cells was achieved in the 0.5%PEIPOS group after 48 h of continuous treatment. This behavior was also dose-dependent (Figure 4(d)). LSC permits the measurement of apoptosis based not only on cell morphology but also on cell staining for different apoptosis indicators, namely YoPro for early apoptosis (Figure 4(e,f) and Supplementary Figure S2(b)). The dye-based analysis shows patterns similar to the morphology-based one, with an increased percentage of cells stained by YoPro in groups 0% and 0.5%PEIPOS treated for 48 h continuously, again in a dose-dependent manner (Figure 4(e,f)).

The cytotoxicity results combined with the imaging analysis indicate PEIPOS liposomal formulations caused an increased β-tubulin stabilization and more apoptotic cells in the population, especially with extended treatment times, confirming that the mechanism of action of PTX loaded in the liposomes remains the same.

### bPEI-modified liposomes can simultaneously complex siRNA with chemotherapeutics for combination treatment of MDR cancer

**Figure 5.**
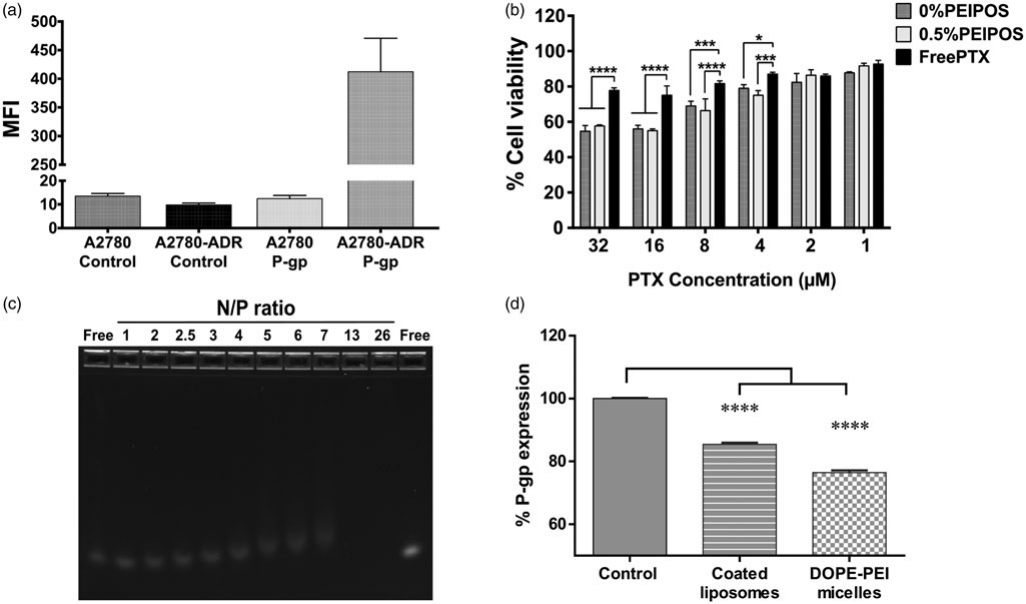
PEIPOS efficacy against MDR ovarian cancer cells and siRNA complexation of surface-modified PEIPOS liposomal formulations. (a) P-gp characterization of the A2780 and A2780-ADR cells using fluorescently-labeled P-gp antibody. The mean fluorescence intensity (MFI) was normalized by isotype control. *n* = 3, mean ± SD. (b) Cytotoxicity of noncoated and coated PEIPOS formulations on A2780-ADR P-gp overexpressing human ovarian cancer cell line following 4 + 44h treatment scheme in serum-complete media. Data shown indicate triplicate mean ± SD from blinded experiment. *p < .05, ***p < .0005, ****p < .0001, two-way ANOVA with Tukey’s multiple comparisons test. (c) Ethidium bromide stained agarose gel electrophoresis of free siRNA and its complexes with 0.5%PEIPOS at different N/P ratios. (d) P-gp downregulation on A2780-ADR cells after treatment with 0.5%PEIPOS formulation with an N/P 13 ratio. The downregulated protein determination was carried out at the end of 48h incubation following 4h of treatment in serum-complete media. *n* = 3 independent trials, mean ± SD.

A2780 and A2780-ADR cells were first characterized by their P-gp expression on the cell membrane. On P-gp overexpressing A2780-ADR cells (Figure 5(a)), coated and noncoated PEIPOS enhanced cytotoxicity when compared to free PTX in concentrations varying from 32 to 4 µM (Figure 5(b)). Despite a better association (Figure 2(b)) and favorable cytotoxicity data with HeLa cells (Figure 5(a)), bPEI-coating did not translate into improved cytotoxicity on A2780-ADR cells (Figure 5(b)), due mostly to the MDR characteristic of the cells. Empty PEIPOS formulations had no significant cytotoxicity on this cell line either.

The siRNA complexation with 0.5%PEIPOS was characterized, along with its stability and downregulation ability. At a fixed concentration of siRNA not fully complexed, the size of the nanosystem increased, and the ZP decreased due to the presence of excess/free nucleic acid and incomplete condensation of the bPEI-chains. With the increasing concentrations of liposomes, and thus the N/P ratio, the complexation of excess siRNA molecules increased, and both size and ZP returned to their approximate initial values (Table 2 and Supplementary Figure S3(a,b)). At an N/P of 13, siRNA is fully complexed through electrostatic interactions between negatively charged siRNA and the positively charged surface-available bPEI (Figure 5(c)). PTX loading into the liposomes did not change the complexation behavior of the liposomes. Moreover, PEIPOS protected the siRNA from *in vitro* RNase degradation (Supplementary Figure S3(c)), which further supports the applicability of these formulations.

**Table 2.** Changes in 0.5% PEIPOS characterization parameters after complexation with scrambled siRNA (siSCR) at various N/P ratios.

Sample	Mean diameter (nm)	PdI	Zeta potential (mV)
0% PEIPOS	164.5 ± 3.77	0.104 ± 0.02	−4.8 ± 0.32
0.5% PEIPOS	172.6 ± 1.83	0.088 ± 0.04	16.0 ± 0.92
0.5% PEIPOS N/P 2	262.3 ± 6.42	0.098 ± 0.08	−14.6 ± 1.40
0.5% PEIPOS N/P 4	225.4 ± 3.10	0.134 ± 0.06	−2.1 ± 0.04
0.5% PEIPOS N/P 6	197.0 ± 1.47	0.132 ± 0.09	2.4 ± 0.36
0.5% PEIPOS N/P 13	180.3 ± 2.75	0.018 ± 0.01	14.6 ± 1.73

Results express the average of *n* = 3 ± SD.

To investigate the ability to downregulate a target protein in MDR cancer cells, PEIPOS were complexed with anti-P-gp siRNA (siMDR1). P-gp is overexpressed 33-fold on A2780-ADR cells compared to their sensitive counterpart (Figure 5(a)), and it is the main resistance mechanism against chemotherapeutics in this cell line. Figure 5(d) shows that significant P-gp downregulation (*p* < .001) with 0.5%PEIPOS and 100 nM siMDR1 was achieved in serum-complete media in a 4 + 44 h treatment scheme, which was very similar to the downregulation obtained by bPEI-DOPE micelles used as positive controls. The results confirm not only the siRNA complexation ability of the bPEI-coated PEIPOS formulations but also the ability to delivery siRNA into the cancer cells in a serum-complete medium, with subsequent gene downregulation.

### *In vivo* efficiency of the co-delivery of paclitaxel and siMDR1

**Figure 6.**
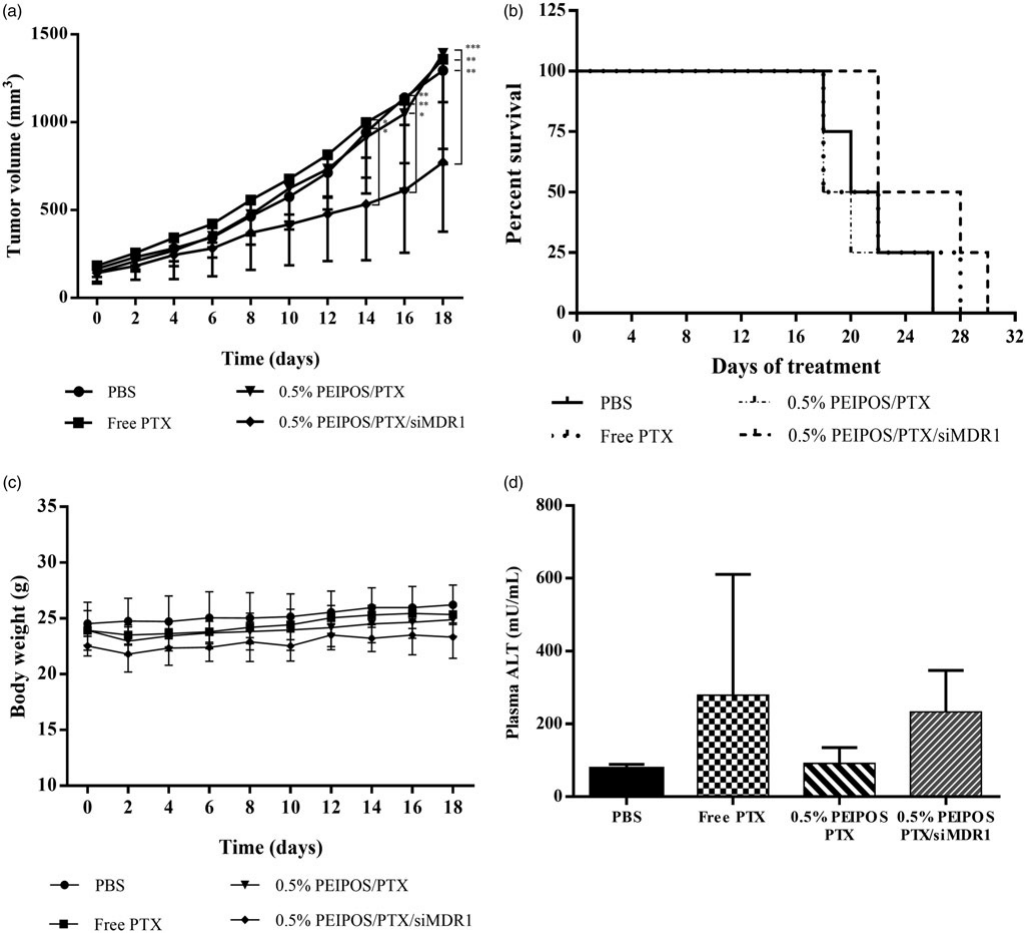
*In vivo* evaluation of the formulation efficacy in nude athymic mice bearing A2780-ADR resistant human ovarian tumor xenografts. (a) Antitumor efficacy of liposomal and nonencapsulated PTX formulations on nude athymic mice bearing A2780-ADR resistant human ovarian tumor xenografts. After tumors were established, mice were treated every other day (first injection on day 0) with saline (•), PTX solubilized in Cremophor (?), 0.5%PEIPOS/PTX (?) or 0.5%PEIPOS/PTX/siMDR1(?) at 5.5 mg/kg of PTX and 0.8 mg/kg of siMDR1. (*n* = 4) Each point represents a mean ± S.D., two-way ANOVA with Dunnett’s multiple comparisons. ****p < .0001 (b) The survival curve of tumor-bearing animals. Mice were treated as indicated in (a) throughout the study period. The survival end-point was when tumors reached 1500 mm^3^. (c) No significant differences were observed in body weight of the animals. (d), Effect of different treatments on liver function evaluated by quantification of alanine aminotransferase (ALT) levels in blood samples collected from the animals before euthanasia. Only a mild increase in ALT levels in animals treated with 0.5% PEIPOS/PTX/siMDR1 was observed, but no statistical difference was identified among the groups.

For the tumor inhibition assay, PTX was used at 5.5 mg/kg, a dose 2–4 times lower than that used in other *in vivo* studies (10–20 mg/kg doses) (Abouzeid et al., 2014; Salzano et al., 2015; Yang et al., 2015). Animals were treated continuously on alternate days until 12 injections were administered, a 66 mg/kg and 9.6 mg/kg cumulative dose of PTX and siMDR1, respectively, or until tumors reached 1500 mm^3^. As observed in Figure 6(a), mice treated with free PTX or without siMDR1 showed no tumor growth inhibition compared to controls, whereas 0.5% PEIPOS/PTX/siMDR1 had tumor volumes approximately 40% smaller than controls 18 days after the treatment started. Tumor growth inhibition was compared between the groups until day 18, where the survival rate of non-siRNA-treated groups and control started decreasing (Figure 6(b)). It is worth noting that, on day 18, two out of four animals in the 0.5% PEIPOS/PTX/siMDR1 group had tumor volumes below 500 mm^3^, which did not occur in any animal of the other groups in this study, which showed volumes above 1000 mm^3^. Additionally, western blot analysis was accomplished to evaluate if the contribution of P-gp downregulation in the tumor growth inhibition effect. P-gp expression in the tumors of animals treated with 0.5% PEIPOS/PTX/siMDR1 showed a 10% decrease in the protein expression compared to the control group treated with PBS (Supplementary Figure S4). This decrease, however, was not statistically different and one explanation for the small difference in protein levels of siMDR1-treated animals could be related to protein recover. Yadav, van Vlerken (Yadav et al., 2009) suggested a reversal in the silencing effect of siMDR1 in resistant ovarian cancer cells SKOV3-TR after 48 h of transfection. Other studies in different cancer cell lines also show a short duration of P-gp knockdown, with recover to initial protein levels two to three days after transfection (Wu et al. 2003; Malmo et al., 2013). Given that tumors were excised from the animals at least 48 h after the last administration of the formulations (or later if tumors had not reached 1500 mm^3^), levels of P-gp might have returned close to normal. It is also valid to mention that, despite the advantage of being able to directly check the protein expression levels, immunoblotting uses total tumor lysate, which also contains nontumor stromal and infiltrating cells, preventing the distinction of P-gp in tumor and normal cells (Mechetner, 2007).

There was no significant variation in animal weight during the treatment period, and ALT levels were not significantly different among the groups (Figure 6(c,d)), confirming the absence of serious toxicity signs during the study. Only a mild increase in the levels of ALT was seen, which is consistent with the presence of nanoparticles in the liver, but do not suggest acute liver damage. However, no statistical difference was identified among the groups.

## Discussion

PEGylation is a well-known strategy for increasing the circulation time of nanoparticles, and it is one of the most exploited surface modification of nanomedicine-based drug carriers for passive targeting via the EPR effect. Doxil^®^, PEGylated liposomal doxorubicin, is one of the most important products that has successfully implemented this strategy. Moreover, in addition to being the first FDA-approved nanomedicine formulation in the market, Doxil^®^ almost single-handedly revived the nanomedicine area that was under great suspicion in the late 1980s (Poste, 1983; Barenholz, 2012). However, PEGylation also reduces the nanoparticle interaction with the tumor cells by hindering the nanoparticle-cell contact. This now-established catch-22 is called the ‘PEG dilemma.’ In addition to poor cellular interaction and internalization, it has been shown that PEGylation affects the rates of transfection, decreases gene silencing, and consequently compromises gene delivery by nanomedicines (Verhoef & Anchordoquy, 2013). Thus, while the benefits of PEGylation remain important, the necessity to find more efficient and versatile options becomes an obvious task. Among the alternatives to PEG for developing long-circulating liposomes are oxazolines ( Gaspar et al., 2014, 2015), poly[*N*-(2-hydroxypropyl) methacrylamide)] (Whiteman et al., 2001), poly-*N*-vinylpyrrolidones (Torchilin et al., 2001) and polyvinyl alcohol (Takeuchi et al., 2001). But the functionality of PEG, or the lack of it, provides a challenge. Modification of the distal ends of the polymers or using different ligand-based approaches to increase the cellular interaction and functionality of the nanocarriers can bring additional problems at each step.

In this study, liposomes were surface-engineered using an in-house synthesized bPEI-lipid conjugate to alter their surface properties. When incubated in serum-containing media, the ZP of all liposomal formulations became negative (Figure 1(a)). This shift could be attributed to the adsorption of albumin and other serum proteins, which are negatively charged at pH 7.4 (Milošev, 2012) onto the liposome surface. The results suggest a steric, rather than electrostatic, stabilization since liposomes with an initial charge, either negative or positive, had a similar amount of adsorbed protein on their surface and the same final ZP. The *in vitro* stability data in an aqueous environment reproducing the osmolarity, salt and protein concentrations found *in vivo* suggests that bPEI could be a suitable substitute for PEG and emphasizes the suitability of such novel formulations for therapeutic applications. Additionally, the lower leakage of surface-modified liposomes upon storage at 4 °C (Table 1) indicates an improved liposomal membrane packing by insertion of DOPE into the bilayer.

In general, *in vitro* drug release testing is used as a characterization parameter for nanomedicine-based drug delivery systems to evaluate the formulation development, the scale-up and the production settings in the quality control chain (Burgess et al., 2004). However, in our study, we evaluated the *in vitro* drug release at different pH conditions (Figure 1(e)) to also investigate this parameter’s relevance to the therapeutic applications and *in vivo* physiological environment that the liposomes would be exposed to. The lower release from liposomes at pH 7.4 suggests that the bPEI-coated liposomes are not ‘leaky,’ meaning that they retain their therapeutic cargo while in the circulation. Furthermore, the coating does not prevent the encapsulated drug from being released, as was the case with PEGylated stealth cisplatin liposomes, which do not release cisplatin either *in vitro* or *in vivo* (Lasic et al., 1999). The proton-sponge effect remains, although challenged recently (Yue et al., 2011; Ur Rehman et al., 2013), one of the main features of PEI-mediated gene delivery. This increased buffering ability could easily disrupt the endosomal membrane with its acidic environment following internalization of the bPEI-coated PEIPOS and provide an effective endosomal escape. An endosomal pH of ∼5.0 is another physiological microenvironment to which PEIPOS would be exposed, and therefore, their release was also assessed in acidic conditions. At pH 5.0, release from PEIPOS was faster than that at neutral pH. Together, the behavior of PEIPOS in both pH conditions, lead to the conclusion that the PEIPOS can retain the majority of the hydrophobic drug in an environment that resembles the circulation and upon reaching the tumor microenvironment, and particularly the target cells, the drug can be released more effectively. The results fully demonstrate the advantages of bPEI-coating as a responsive alternative to conventional PEGylation.

Cellular association experiments suggest a positive correlation between the amount of bPEI-PE present in the liposome surface and the cell interaction (Figure 2). PEIPOS 0.5% was the most highly cell-associated formulation at almost double the 0.1%PEIPOS level. It is important to note that surface-engineered liposomes interacted at a significantly higher level even with the drug-resistant ovarian cancer cells in serum-complete medium. The results suggest that the increase in the cellular association cannot be tied solely to the surface charge of the liposomes since in serum-complete media, the overall charge of the PEIPOS drops to negative values. Despite the overall negative charge, locally available bPEI chains still increased the cellular association of the attached carrier. Similar results were also reported with PEI-coated mesoporous silica nanoparticles (MSNP) loaded with PTX (Xia et al., 2009). This can be explained by the possibility of specific ligand-receptor like interactions between the PEI and cell surface, mainly by the sulfated proteoglycans, as reported by Hess et al. (2007). bPEI-coating on our liposomal formulations provides the important advantage of functionality over conventional PEGylation and could be a highly viable option for preparation of non-PEGylated, yet stable nanocarriers. In agreement with these results, the incubation of 0.5% PEIPOS with cells at 4 °C and/or pretreated with bPEI also indicate a receptor-based interaction (Figure 2(d)).

In addition to increased cancer cell association, protein adsorption onto the PEIPOS surface has another unexpected but important advantage. Opsonization of nano-sized carriers and their subsequent capture by macrophages starts with the recognition of the carrier by receptors or binding sites on the membrane of endocytic cells. If liposomes coated with a protein, such as albumin and globulins in our case, are simultaneously present in an environment which has excess of the same protein, the capture of liposomes can be decreased due to preliminary saturation of the binding sites on macrophages for those proteins (Torchilin et al., 1980). Thus, the longevity in the circulation can be increased. Torchilin et al. (1980) elegantly showed that the albumin- and ɣ-globulin-coated liposomes were captured 20% and 25% less by the macrophages, respectively, and that this phenomenon was free protein concentration-dependent. Moreover, Ehrenberg *et al.* showed that the capacity of nanoparticle surfaces to adsorb protein is an indication of their cellular association and that cellular interaction is not dependent on the adsorbed protein type (Ehrenberg et al., 2009). It should be noted that the protein corona formed on the positively-charged nanoparticles is retained during the cellular uptake and can protect the cells from possible damage until it becomes degraded in the lysosomes (Wang et al., 2013), which is supported by our cytotoxicity results discussed later.

Penetration into solid tumors is one of the current challenges affecting successful cancer therapy in the clinic. However, it has also been an area underestimated that has gone largely uninvestigated in many studies (Minchinton & Tannock, 2006). Following the monolayer studies, penetration of PEIPOS in the spheroid model was evaluated (Figure 3). The combined results of flow cytometry and CLSM analysis indicate that bPEI coating not only helps to increase cellular association of the liposomes with cells in monolayers, but it also allows higher penetration and interaction of liposomes with cells in 3D tumor spheroid models. Results confirm that 0.5%-bPEI-modified liposomes penetrate the 3D tumor mass-mimicking models by bypassing barriers including physical penetration and decreased cellular interaction in a highly heterogeneous environment. This penetration does not interfere with or diminish the enhanced association of the bPEI-modified liposomes with the individual cells in all layers (Figure 3(a)) and is not subject to a penetration ‘barrier effect.’ While the penetration profile analyses were carried out up to 250 µm depth (approximately radius of the spheroids), the meaningful signals from 0.5% PEIPOS went only as deep as 100 µm. It should be noted that the signals from the core areas of the spheroids would not be collected due to faltered laser penetration after ∼70 µm (Pampaloni et al., 2007; Verveer et al., 2007; Carver et al., 2014). MFI Z-projections are created by using the maximum value of each pixel over all stacks at the particular pixel location. Thus, they emphasize the differences of rhodamine intensity.

The administration of PTX in liposomal carriers instead of its conventional, Cremophor EL^®^/ethanol-based formulation (Taxol^®^) is highly desirable. By liposomal delivery of highly hydrophobic PTX, the severe toxicity of the formulation components, caused mainly by the surfactant, can be avoided (Gelderblom et al., 2001). Moreover, all-biocompatible liposomal carriers can be easily modified and targeted to improve tumor specificity and decrease systemic side effects (Deshpande et al., 2013). On the other hand, when PTX is loaded into the liposomal formulations, its association with the carrier delays intracellular PTX release, which can decrease the immediacy of the drug’s action. This behavior was not observed on HeLa cells, with enhanced cytotoxicity of bPEI-coated PTX liposomes equivalent to the free drug (Figure 4(a)). The data confirm the improved cell association promoted by bPEI coating. The lack of cytotoxicity of empty liposomes also supports the safety of the carrier system achieved for two main reasons. First, the low molecular weight bPEI used has significantly less cytotoxicity against mammalian cells. And second, we performed the experiments in a serum-complete medium so that the overall positive charge of the nanosystem was shielded by adsorbed proteins on the surface.

The cell cycle distribution was analyzed by LSC (Figure 4(c–f) and Supplementary Figure S2), which combines the advantages of flow cytometry with the ability to evaluate cell morphology and quantification of cell components depending on the different fluorescent dyes utilized in the assay (Luther et al., 2004). Together, the findings from both approaches indicate an advantage of continuous treatment with PTX instead of a pulsed regimen of 4 + 44 h for HeLa cells. In accordance with the LCS analysis, the CLSM results regarding the β-tubulin polymerization indicate that the encapsulation of PTX into liposomal carriers surface-engineered with bPEI-lipid does not change the course of action of PTX. Moreover, as supported by the cytotoxicity results, the PEIPOS do not interfere with the PTX mechanism of action. Altogether, these findings further uphold the overall advantages of these reported novel liposomal formulations.

An enhanced cytotoxic effect of coated or noncoated PEIPOS in A2780-ADR cells was also observed (Figure 5(b)) and underlies the advantages of nanomedicine-based treatments. While free drugs, including PTX, enter the cells through passive diffusion or active transporters, nanocarrier internalization frequently involves endocytic pathways (Torchilin, 2005). P-gp affinity for PTX is 100 times higher when the P-gp protein is in the lipid bilayer than in a detergent (Jin et al., 2012). This allows the protein to act as a ‘vacuum cleaner’ sweeping out drug molecules as they diffuse through the bilayers. Encapsulation of PTX in liposomes enhances the endocytosis of the whole system and helps in part to overcome this P-gp efflux activity, (Torchilin 2005) resulting in higher cytotoxicity compared to free drug. However, the reduced cytotoxicity of PTX even with bPEI-coated liposomes emphasizes the challenges of monotherapy of resistant cancers and the importance of combination therapy approaches.

Versatility of novel formulations for combination therapy is very important, and the systems should be adaptable for different applications and purposes. The bPEI-lipid conjugates, mainly bPEI-PE, complexes siRNA and transfects a wide range of cells both *in vitro* and *in vivo* (Navarro et al., 2012, 2014; Essex et al., 2015). Thus, we evaluated the ability and limits of the bPEI-coated liposomes (0.5%PEIPOS) as potential carriers of nucleic acids into MDR cancer cells (Figure 5), that could not be achieved by conventional PEGylation without further surface modification. The successful downregulation of P-gp via siMDR1 confirms the overall hypothesis that the complexed and protected siMDR1 are efficiently internalized with PEIPOS by MDR cells despite the overall negative charge due to protein adsorption on the liposomal surface and is also able to escape possible endosomal encapsulation due to the ‘proton-sponge’ effect of bPEI chains and be released into the cytoplasm to trigger RNAi.

In the *in vivo* study, there was an absence of effect in PTX-treated groups other than that associated with siRNA (0.5% PEIPOS/PTX/siMDR1) (Figure 6(a)). The lack of effect may be attributed to the low-dose regimen used in this study. It is hypothesized that when a low dose is used, coupled with the fact that A2780-ADR cells overexpress P-gp and were thus expected to show resistance to PTX, it was not sufficient to promote tumor growth inhibition, even with prolonged drug administration. However, such a dose regimen allowed the observation of a synergistic effect of associated siMDR1 and PTX. This synergism is likely to have been masked by the use of higher doses of PTX. RNA interference has been studied and explored for the past 20 years and been shown to have great potential in cancer therapy due to the specificity related to gene silencing ( Zhou et al. 2014; Kim et al., 2016; Tatiparti et al., 2017). Given the versatility of the formulation developed in this study, particularly its ability to carry siRNA and PTX simultaneously, the experimental design was intended to co-administer both approaches throughout the experiment. Anti-P-gp siRNA was chosen for this study for targeted gene therapy since it is both involved in MDR of various tumors and in view of the feasibility for evaluation of treatment response. The association of siMDR1 with PTX, which is a potent drug often used in the clinic, allowed the evaluation of the capacity of 0.5% PEIPOS to successfully deliver both payloads that led to a successful *in vivo* outcome.

## Conclusions

In this study, we developed novel low-molecular-weight bPEI-coated liposomal formulations, PEIPOS that can be used not only as drug delivery systems, but also as short oligonucleotide carriers. This approach leads to the combination of both types of payload in a single delivery system for improved outcomes. We showed that bPEI-coated PEIPOS formulations have favorable stability properties in serum protein-containing environments. Moreover, the protein adsorption onto this system may act as a shield against macrophage uptake and facilitate cellular biocompatibility. Further pharmacokinetic and biodistribution studies would be necessary to confirm this assumption. The PEIPOS formulations improved the cellular association drastically and also enhanced the cytotoxicity without interference with the mode of action of its cargo chemotherapeutic. The formulations also improve the penetration into 3D tumor-mimicking models, overcoming another important challenge by delivering drugs into deeper layers of solid tumors. The co-loaded formulation carrying siMDR1 and PTX used *in vivo* promoted tumor growth inhibition in an MDR xenograft ovarian tumor model. This effect was not observed when only PTX was administered, even in bPEI-modified liposomes, highlighting the potential advantages of combining gene therapy and chemotherapy to overcome drug resistance. Here, we documented the concept of non-PEGylated, yet stable, functional surface-engineered novel liposomal formulations that possess a long-circulating property when low molecular weight bPEI is substituted for PEG. *In vivo* studies, in addition to the ones presented here, are needed to further investigate the potential of these nanopreparations, optimize their dose and administration regimen to improve the therapeutic response. Overall, the safety and efficacy reported for PEIPOS formulations in this study make them as a promising alternative to PEG and as drug delivery system for combination therapy to treat cancer.
